# SARS-CoV-2 Transmission from Human to Pet and Suspected Transmission from Pet to Human, Thailand

**DOI:** 10.1128/jcm.01058-22

**Published:** 2022-10-31

**Authors:** Chutchai Piewbang, Panida Poonsin, Pattiya Lohavicharn, Sabrina Wahyu Wardhani, Wichan Dankaona, Jiratchaya Puenpa, Yong Poovorawan, Somporn Techangamsuwan

**Affiliations:** a Department of Pathology, Faculty of Veterinary Science, Chulalongkorn University, Bangkok, Thailand; b Animal Virome and Diagnostic Development Research Group, Faculty of Veterinary Science, Chulalongkorn University, Bangkok, Thailand; c The International Graduate Course of Veterinary Science and Technology (VST), Faculty of Veterinary Science, Chulalongkorn University, Bangkok, Thailand; d Department of Pediatrics, Faculty of Medicine, Center of Excellence in Clinical Virology, Chulalongkorn University, Bangkok, Thailand; Jockey Club College of Veterinary Medicine

**Keywords:** COVID-19, SARS-CoV-2, animal, cat, coronavirus, zoonotic infections

## Abstract

Coronavirus disease 2019 (COVID-19), caused by severe acute respiratory syndrome coronavirus 2 (SARS-CoV-2), has been the cause of human pandemic infection since late 2019. SARS-CoV-2 infection in animals has also been reported both naturally and experimentally, rendering awareness about a potential source of infection for one health concern. Here, we describe an epidemiological investigation of SARS-CoV-2 infection in 639 cats and 224 dogs throughout multiple waves of COVID-19 outbreaks in Thailand. To indicate the potential source of infection, we performed SARS-CoV-2 genomic sequencing of samples obtained from pets and contacted humans, combined with in-depth interviews to support the epidemiological investigation. In the tested animals, SARS-CoV-2 RNA was present in 23 cases (19 cats and 4 dogs). Whole-genome sequencing of selected samples showed various SARS-CoV-2 variants of concern, which included the original European lineage (B.1), Alpha (B.1.1.7), Delta (B.1.617), and Omicron (BA.2). Among SARS-CoV-2-positive pets, 34.78% had evidence of contact with infected humans. Together with genomic analysis and an overlapping timeline, we revealed evidence of viral transmission from infected humans as the primary source, which spread to household cats via an undefined mode of transmission and most likely circulated between cohoused cats and caretakers within the weeks before the investigation. The SARS-CoV-2 surface glycoprotein (spike gene) obtained from caretakers of individual cats contained sequence signatures found in the sequences of infected cats, indicating possible exposure to the virus excreted by cats. Although pet-to-human transmission of SARS-CoV-2 is considered relatively rare, our study provides suspected episodes of human infection from animals that were initially infected through contact with infected humans.

## INTRODUCTION

Severe acute respiratory syndrome coronavirus 2 (SARS-CoV-2) was first identified in humans presenting with progressive pneumonia in Wuhan, China, in late December 2019 and subsequently spread across the world, causing the global coronavirus disease 2019 (COVID-19) pandemic (1). The origin of this emergent virus in humans is still questioned; however, it is possibly related to wild animals being sold in a live-animal market in Wuhan, where the first outbreak was reported (2). Because previous SARS-CoV-1 emergence has been proven to be related to animal origins, intensive investigations of SARS-CoV-2 in various animal species have been conducted, resulting in the identification of coronavirus, which is genetically closely related to SARS-CoV-2, in bats and pangolins (3–5). These findings indicate the possible roles of animals as reservoirs for SARS-CoV-2. Information about a specific cellular receptor, angiotensin-converting enzyme 2 (ACE2), for SARS-CoV-2 infection has been identified in many animal species (6, 7); hence, infection of other animals by this virus is possible, and infections have been reported (as of 30 April 2022) in animals (8) such as cats, dogs, minks, otters, pet ferrets, lions, tigers, pumas, snow leopards, gorillas, white-tailed and mule deer, fishing cats, binturongs, coatimundis, spotted hyenas, Canada lynx, hippopotamuses, hamsters, giant anteaters, West Indian manatees, and marmosets in various countries (8). Although natural infection by SARS-CoV-2 in animals has resulted mostly from close contact between susceptible animals and infected owners or animal caretakers living in the same household (9, 10), animal-to-animal and animal-to-human transmission of SARS-CoV-2 has recently been reported in infected farmed minks in the Netherlands (11, 12). Although the public health risk of exposure to SARS-CoV-2-infected animals has been considered to be low, awareness of potentially zoonotic involvement in the SARS-CoV-2 epidemic is needed and requires intensive focus.

In Thailand, humans have been facing the COVID-19 pandemic over five waves since January 2020, resulting in over 4 million people being infected and more than 30,000 deaths related to SARS-CoV-2 infection (13). In terms of disease control and prevention, extensive surveillance systems, not only for humans but also for animal interventions, have been established. Due to close contact between owners and pets, mainly cats and dogs, the risk of exposure to SARS-CoV-2 infection from infected owners to their pets is highly concerning (10, 14). On the other hand, SARS-CoV-2 spillover from contacted animals to susceptible humans, even if it is currently determined to be low, should not be underestimated.

As part of the Thai national surveillance effort, we conducted surveillance of SARS-CoV-2 infection in cats and dogs residing in central and rural areas of Thailand through five waves of COVID-19 outbreaks by collecting swab samples from sheltered, hospitalized, and outpatient cats and dogs from January 2020 to May 2022. Owners, veterinarians, and pet caretakers who had contact with SARS-CoV-2-infected cats and dogs were enrolled in this study. Here, we describe investigations of SARS-CoV-2 infection in cats and dogs in Thailand, combining epidemiological information and whole-genome sequencing of the detected virus. Furthermore, SARS-CoV-2 RNA sequences obtained from infected cats revealed evidence of transmission from infected humans as the initial infection followed by transmission between cohoused cats. The presence of the viral RNA obtained from subsequent pet caretakers indicated possible exposure to the virus excreted by cats.

## MATERIALS AND METHODS

### Ethical statement.

This study was approved by the Institutional Animal Care and Use Committee (IACUC) of the Faculty of Veterinary Science, Chulalongkorn University (approval no. 2131001) and the Institutional Review Board (IRB) of the Faculty of Medicine, Chulalongkorn University (approval no. 178/64). All methods were carried out in accordance with relevant guidelines and regulations. The study complies with the ARRIVE guidelines.

### Investigation timeline and geographic distribution.

**FIG 1:**
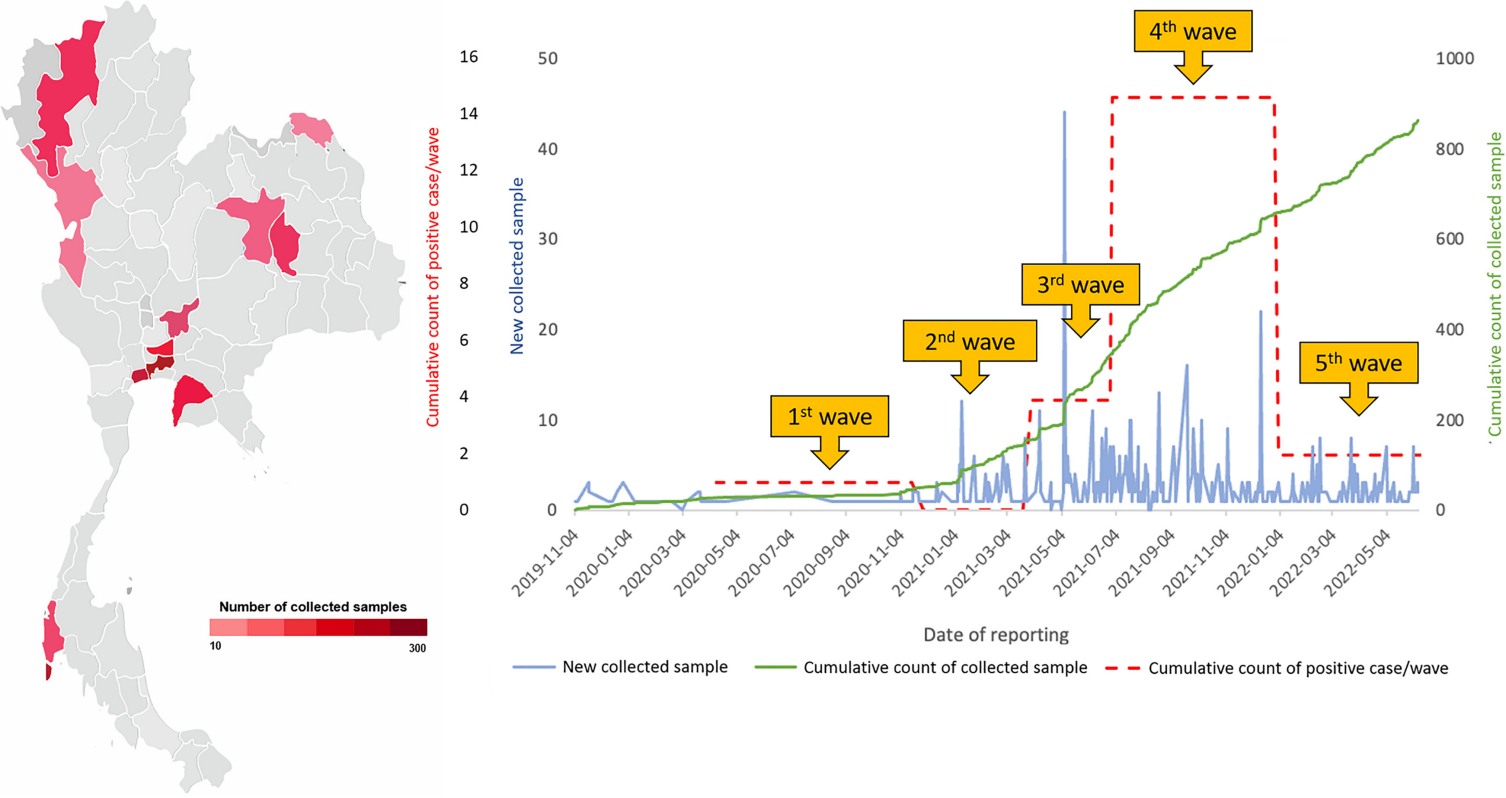
Geographic and schematic overviews of sample collection and SARS-CoV-2-positive samples. Shown are the geographic distribution of samples collected in this study (left) and the total numbers of samples collected during the first to fifth COVID-19 waves in Thailand (right). The number of collected samples in each region is labeled according to a red color range that corresponds to the graph in panel B (blue line).

According to the official announcement of the Ministry of Public Health regarding the first case of local infection in January 2020 and the report of Thailand’s Centre for COVID-19 Situation Administration (CCSA) concerning the first wave of the COVID-19 pandemic in Thailand in March 2020 (15), we initially investigated SARS-CoV-2 infection in cats and dogs that were either sheltered or brought to animal hospitals located in the Bangkok (BKK), Phuket (PK), Chonburi (CB), and Tak provinces, which were either areas where the disease was endemic or areas under state quarantine, beginning on 24 March 2020. Subsequently, the investigated areas were extended for sampling following the second to fifth waves of COVID-19 outbreaks in Thailand, comprising the Pathum Thani, Samutsakorn (SSK), Bueng Kan, Khon Kaen, Chiang Mai, Saraburi, and Krabi provinces, from 1 December 2020 to 23 May 2022. We also tested swab samples collected from cats and dogs since 1 November 2019 to trace back the presence of SARS-CoV-2 before the official announcement of COVID-19 emergence. The geographic distribution of the collected samples is illustrated in Fig. 1.

### Animals and sample collection.

We investigated the presence of SARS-CoV-2 in 863 animals, consisting of 639 cats and 224 dogs, that had been sheltered, hospitalized, or brought to animal hospitals/clinics. Samples from animals enrolled in this study were collected upon available owner consent. The samples were collected from oropharyngeal and/or nasal swabs using a disposable sterile sampling kit (Shanghai Dediag Biotechnology Co. Ltd., Shanghai, China) following approval by the U.S. Food and Drug Administration (FDA). The collected swabs were immediately placed on ice and transported in coolers to a laboratory of the Animal Virome and Diagnostic Development (AVDD) Research Group, Faculty of Veterinary Science, Chulalongkorn University, Thailand.

Essential signalments and clinical presentations were recorded at the time of sampling. If a positive case was present, further sample collection was performed on other cats and dogs residing in the same household or shelter. Owners, veterinarians, and pet caretakers who had been involved with SARS-CoV-2-positive pets were asked for permission to conduct a trace investigation by providing personal information, SARS-CoV-2 antigen testing status, and nasopharyngeal swab samples according to their convenience and safety criteria in accordance with CCSA recommendations.

### Nucleic acid extraction and detection of SARS-CoV-2.

Swab samples were subjected to RNA extraction using automated nucleic acid isolation and purification (QIAcube Connect; Qiagen, Hilden, Germany). Briefly, a 140-μL supernatant sample was obtained and subsequently mixed with AVE solution containing carrier RNA, which was provided with the QIAamp viral RNA extraction kit (Qiagen, Hilden, Germany) according to the manufacturer’s recommendations. An internal process control for RNA extraction was added prior to extraction. The mixtures were placed on the automated extraction machine with the settings suggested by the manufacturer, resulting in 60 μL of RNA eluent from the extraction process. The quality and quantity of the extracted RNA were determined by a spectrophotometer at an absorbance of 260/280 nm.

SARS-CoV-2 RNA detection was performed using probe-based quantitative real-time reverse transcription-PCR (qRT-PCR) with multiple primers targeting the N1 and N2 genes and the RNA-dependent RNA polymerase (RdRp) gene according to U.S. Centers for Disease Control and Prevention (CDC) (16) and WHO (17) recommendations, respectively. The qRT-PCR assay was performed using the QuantiNova probe RT-PCR kit (Qiagen, Hilden, Germany) according to the manufacturer’s recommendations. Briefly, the 20-μL reaction mixture contained 10 μL of 2× QuantiNova probe RT-PCR master mix, 0.2 μL of 100× QuantiNova RT mix, 20× primer-probe mix containing 4 μM TaqMan probe and 8 μM each primer, 2 μL of the RNA template, and RNase-free water. Thermal cycling conditions comprised 45°C for 10 min for the RT step, followed by an initial denaturation step at 95°C for 5 min, 40 cycles of 95°C, 5 s of denaturation at 60°C, and 30 s for the annealing and extension processes. Samples that presented threshold cycle (*C_T_*) values of <36, 36 to 40, and >40 were considered positive, suspected positive, and negative, respectively (18). The positive and suspected positive samples obtained from the qRT-PCR results were subsequently amplified for the surface (S), E, RdRp, and N genes using gel-based conventional RT-PCR and bidirectional Sanger sequencing to confirm the presence of SARS-CoV-2. Serum samples were collected from SARS-CoV-2 PCR-positive cats (together with the collection of sera from cohoused cats) at the same time as when the swab samples were further tested for SARS-CoV-2 antibody using the surrogate neutralization test (GenScript Biotech, Jiangsu, China) (Potjanee Srimanote, personal communication).

### Genetic characterization of SARS-CoV-2.

The qRT-PCR-positive samples yielding *C_T_* values of <32 were further characterized for the genetic diversity of the detected SARS-CoV-2 using next-generation sequencing (NGS). In this study, we used the Celemics comprehensive respiratory virus panel (CRVP) (Celemics Inc., Incheon, South Korea) for the sequencing and identification of full-length SARS-CoV-2 genomes. Briefly, 25 ng of extracted RNA was mixed with 5× RNA fragment buffer mix for RNA fragmentation, and subsequently, first-strand cDNA was constructed using 1st-strand synthesis master mix. The first-strand cDNA was subjected to the construction of double-stranded cDNA using 2nd-strand synthesis-1 mix by incubation at 16°C for 60 min, followed by mixing with 2nd-strand synthesis-2 mix at 25°C for 15 min. The double-stranded cDNA was cleaned and repaired, and poly(A) tail oligomers were added using 5× ERA buffer mix. The mixtures were incubated at 4°C for 1 min, followed by multiple incubation steps, including incubation at 20°C for 30 min and 65°C for 30 min, and then placed on ice immediately. The A-tailed DNA was ligated with a ligation reaction mix containing adaptors at 20°C for 15 min. The ligated DNA was purified using CeleMag cleanup beads and subsequently amplified, and the adaptor-ligated library was constructed using CLM polymerase and UDI primers according to the manufacturer’s suggestions. The constructed DNA library was examined for quantity and quality using automated capillary gel electrophoresis (QIAxcel; Qiagen, Hilden, Germany) to confirm the presence of a 200- to 400-bp constructed DNA. Completed DNA libraries were subjected to NGS using the Illumina NextSeq 500 system and the mid/high-output kit v2.5 (300 cycles). The generated FASTQ data were obtained, trimmed, assembled, and analyzed using the Celemics Virus Verifier pipeline, which can identify and generate consensus sequences for the SARS-CoV-2 genome. Nucleotide gaps present in the assembled SARS-CoV-2 FASTQ sequences were filled with nucleotide sequences obtained by conventional RT-PCR-derived Sanger sequencing according to the designed primers specific to the gaps. Furthermore, we attempted to characterize the full-length genome sequence of the SARS-CoV-2 S gene in the positive cases (both animals and their related human subjects) showing low *C_T_* values using multiple conventional RT-PCRs according to previously described primers and protocols (19).

### Lineage classification and phylogenetic analysis of SARS-CoV-2.

The obtained full-length genome sequences of SARS-CoV-2 were subjected to lineage classification using Phylogenetic Assignment of Named Global Outbreak Lineages (PANGOLIN) according to specific amino acid mutations throughout the SARS-CoV-2 sequences (20). Genetic variants were determined according to WHO SARS-CoV-2 variant classifications to determine variants of concern (VOCs). Phylogenetic analysis was completed by comparing nucleotide substitution changes to the 99 whole-genome sequences of SARS-CoV-2 obtained from infected humans and infected pets in Thailand and other pets from around the world, available in the GenBank and GISAID databases. The obtained SARS-CoV-2 sequences were trimmed and aligned using the BioEdit and MAFFT programs, respectively, prior to submission to construct the phylogenetic tree using MEGA 10. The phylogenetic tree was constructed using the maximum likelihood model as a general time reversible with gamma distributed and invariant sites (GTR+G+I) model according to the find-best-fit model used for model selection, with 1,000 bootstrap replicates. The obtained phylogenetic tree was visualized and labeled using iTOL version 6.0 (https://itol.embl.de/) (21). Phylogenetic analysis based on full-length genome sequences of the S gene of SARS-CoV-2 obtained from the contacted humans and infected animals was additionally performed to determine the possible genetic relationships among individual positive cases. The SARS-CoV-2 sequences in the GenBank database obtained from humans and pets at the same time and from the same geographic area (province) were retrieved and used for the analysis of phylogenetic relationships.

### Selection pressure analysis.

Rates of substitution of SARS-CoV-2 S genes obtained from infected animals and their related humans in this study, together with SARS-CoV-2 genomes of humans and pets in Thailand retrieved from the GenBank and GISAID databases, were estimated using nonneutral selection. To determine nonneutral selection, fixed-effects likelihood (FEL) (22) and mixed-effects model of evolution (MEME) (23) methods were used. These methods were implemented via the Hyphy software package (24). Settings and statistical analysis (*P* = 0.1 with a Bayes factor of 50 for the estimation of the rates of nonsynonymous substitutions [*dN*] and synonymous substitutions [*dS*] within each codon) were performed as previously described (25). The rates were calculated as the *dN*/*dS* ratio using the maximum likelihood phylogenetic reconstruction platform according to the general reversible nucleotide substitution model available on the Datamonkey Web server (26). *dN*/*dS* ratios of >1, 1, and <1 were used to define positive selection, neutral mutations, and negative selection, respectively.

### Data availability.

The data that support the findings of this study are available in this article. Ten full-length genome sequences of SARS-CoV-2 were submitted to the NCBI database under GenBank accession no. ON966106 to ON966115, and the obtained S gene sequences were submitted to GenBank under accession no. ON965806 to ON965809.

## RESULTS

### SARS-CoV-2 investigation.

**FIG 2:**
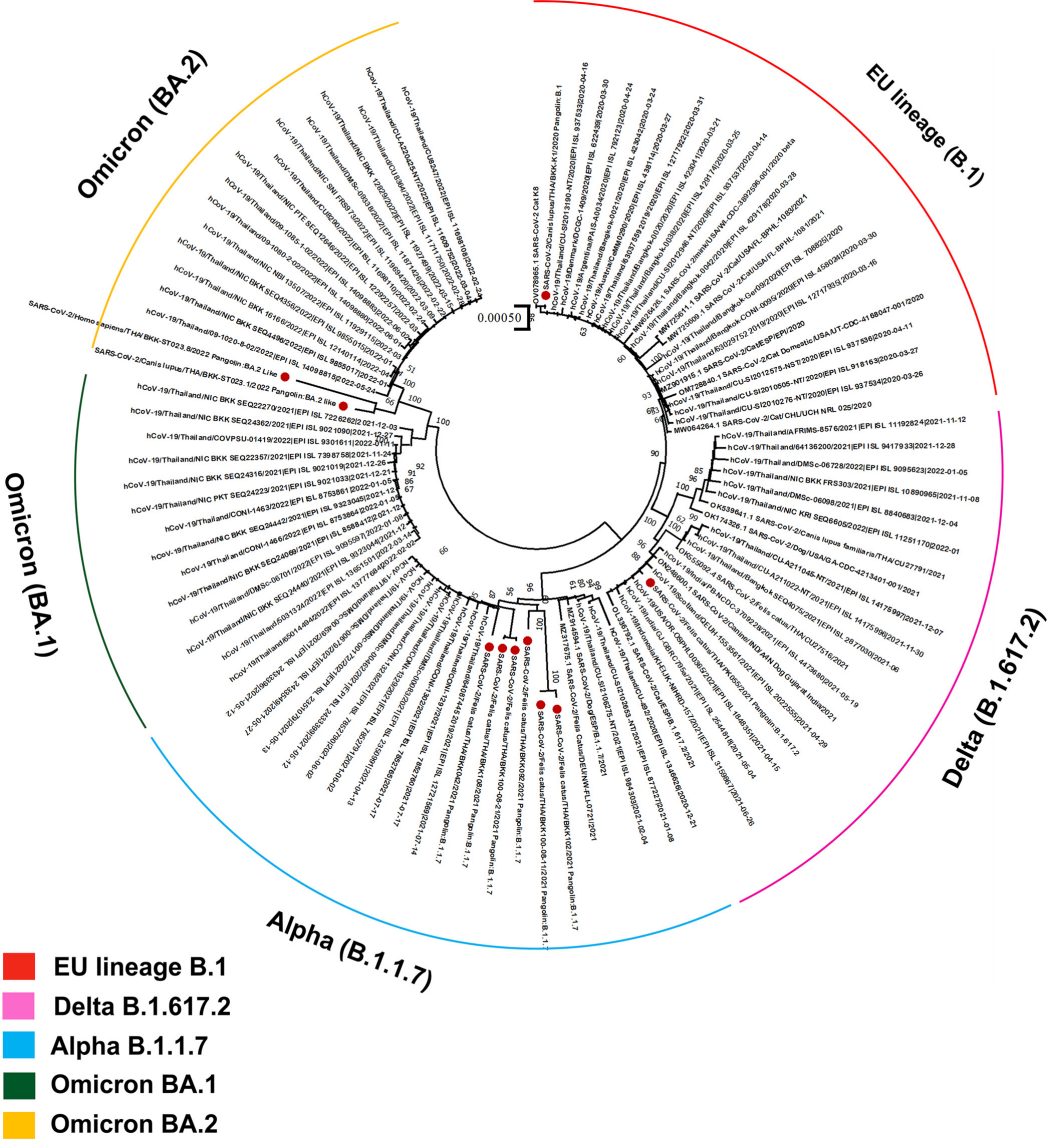
Phylogenetic analysis of SARS-CoV-2 sequences from humans in Thailand from April 2020 to June 2022 and sequences obtained from previously reported animals using the maximum likelihood method. Whole-genome sequences derived from SARS-CoV-2-positive animals from this study are indicated by red dots and are clustered within various VOCs of human SARS-CoV-2 sequences, as indicated by the different colors. The scale bar of 0.00050 indicates units of substitutions per site.

A total of 23/863 (2.67%) samples derived from 19 cats and 4 dogs were positive for SARS-CoV-2 by qRT-PCR. The highest number of positive cases was obtained from Bangkok (BKK) (*n* = 13), followed by Phuket (PK) (*n* = 5), Chonburi (CB) (*n* = 2), Pathumthani (PT) (*n* = 2), and Samutsakorn (SSK) (*n* = 1). Samples collected from cats and dogs in other provinces (Tak, Bueng Kan, Khon Kaen, Chiang Mai, Saraburi, and Krabi) were negative for SARS-CoV-2 by qRT-PCR. The rates of positive SARS-CoV-2 detection were highest in the fourth wave of COVID-19 in Thailand (Fig. 1). The clinical signs of positive animals were mostly asymptomatic infections (56.52%; 13/23); mild respiratory distress, including sneezing and coughing (26.09%; 6/23); and severe pneumonia (13.04%; 3/23). Eight qRT-PCR-positive animals (34.78%), comprising 6 cats and 2 dogs, had evidence of contact with SARS-CoV-2-positive humans. Details of positive cases are described in Table 1. Regarding the samples that presented optimum *C_T_* values (≤35) for whole-genome characterization using Illumina sequencing, full-length SARS-CoV-2 genomes were obtained from 7 samples derived from 6 cats (PK055, BKK042, BKK092, BKK100 [2 samples at different time points], BKK102, and BKK108), 2 dogs (BKK-K1 and BKK-ST023.1), and 1 human (BKK-ST023.8). Phylogenetic analysis-based complete genome sequences revealed that the sequences obtained from cats and dogs were separately clustered into a group of SARS-CoV-2 sequences originating from humans. Similar to the findings with the phylogenetic tree, analysis based on the PANGOLIN pipeline segregated the SARS-CoV-2 sequences presented in this investigation into 4 distinct clades, which were considered to be Alpha B.1.1.7 (*n* = 4), European lineage B.1 (*n* = 1) and derivative B.1.1 (*n* = 2), Delta B.1.617.2 (*n* = 1), and Omicron BA.2 (*n* = 2) VOCs (Fig. 2). Regarding the amino acid substitutions of the spike protein, we also found variants within the regions of the receptor binding domain (RBD) (W535C, K417N, L452R, T478K, and N501Y) and the heptad repeat (HR) (H982A), as shown in Table 2.

**TABLE 1:** Demographic data of SARS-CoV-2-positive cats and dogs with a history of contact with positive humans^*i*^

Sample ID	Host	Sampling date (day mo yr)	Sex	Human lifestyle or animal living habitat	SARS-CoV-2 detection		Respiratory symptom^*f*^	History of contact with positive humans^*g*^
					qRT-PCR (result, *C_T_* value)^*h*^	Antibody		
BKK042	Cat	9 June 2021	M	Indoor	+, 25	−	Healthy	No
BKK092	Cat	5 Aug 2021	M	Indoor	+, 27	+	Cough	Yes
BKK095	Cat	9 Aug 2021	M	Indoor	+, 26	−	Healthy	No
BKK100	Cat	11 Aug 2021	M	Indoor	+, 18	−	Sneezing	Yes
		21 Aug 2021			+, 29	−	Sneezing	No
BKK102^*a*^	Cat	21 Aug 2021	M	Indoor	+, 30	−	Healthy	Yes
BKK103^*a*^	Cat	21 Aug 2021	F	Indoor	−	−	Healthy	Yes
BKK104^*a*^	Cat	21 Aug 2021	F	Indoor	−	−	Healthy	Yes
BKK106^*a*^	Cat	21 Aug 2021	M	Indoor	−	−	Healthy	Yes
BKK107^*a*^	Cat	21 Aug 2021	F	Indoor	+, 36	+	Healthy	Yes
BKK108^*a*^	Cat	21 Aug 2021	M	Indoor	+, 31	+	Healthy	Yes
BKK109^*a*^	Cat	21 Aug 2021	F	Indoor	−	+	Healthy	Yes
BKK-P1^*a*^	Human	21 Aug 2021	F	Single living	+, 31	NA	Sore throat	No
BKK-P2^*a*^	Human	21 Aug 2021	F	Single living	+, 33	NA	Cough	No
BKK-P3^*a*^	Human	21 Aug 2021	F	Single living	+, 34	NA	Healthy	No
BKK-ST10^*a*^	Cat	7 Sept 2021	F	Indoor	+, 34	−	Sneezing	Yes
BKK225	Cat	28 Mar 2022	F	Indoor	+, 34	NA	NA	NA
PK051^*b*^	Cat	27 July 2021	F	Indoor/outdoor	+, 32	−	Sneezing	No
PK053^*b*^	Cat	27 July 2021	F	Indoor/outdoor	−	+	Healthy	No
PK055^*c*^	Cat	28 July 2021	F	Indoor	+, 31	−	Healthy	No
PK057^*c*^	Cat	7 Aug 2021	M	Indoor/outdoor	+, 34	+	Healthy	No
PK058^*c*^	Cat	10 Aug 2021	M	Indoor/outdoor	+, 36	−	Healthy	No
PK060^*d*^	Cat	10 Aug 2021	F	Indoor/outdoor	−	+	Healthy	NA
PK061^*d*^	Cat	10 Aug 2021	F	Indoor/outdoor	+, 35	−	Healthy	NA
SSK004	Cat	28 May 2021	M	Outdoor	+, 33	−	Healthy	NA
CB007	Cat	22 Apr 2021	M	Indoor/outdoor	+, 33	−	Pneumonia	No
CB009	Cat	3 May 2021	F	Indoor/outdoor	+, 35	−	Pneumonia	No
PT027	Cat	5 July 2021	M	NA	+, 36	−	Healthy	NA
PT030	Cat	5 July 2021	M	NA	+, 34	−	Healthy	NA
BKK-ST008	Dog	14 July 2021	M	Indoor/outdoor	+, 33	NA	Cough	Yes
BKK-ST017	Dog	12 Nov 2021	M	Indoor	+, 32	NA	Cough	No
BKK-ST023.1^*e*^	Dog	25 Mar 2022	M	Indoor/outdoor	+, 29	NA	Healthy	Yes
		26 Mar 2022			+, 31	NA	Healthy	
		27 Mar 2022			+, 35	NA	Healthy	
		28 Mar 2022			+, 37	NA	Healthy	
		29 Mar 2022			+, 38	NA	Healthy	
		30 Mar 2022			−	NA	Healthy	
		1 Apr 2022			−	NA	Healthy	
BKK-ST023.8^*e*^	Human	24 Mar 2022	F	Single living	+, 18	NA	Cough	No
		30 Mar 2022			+, 30	NA	Cough	
		3 Apr 2022			+, 37	NA	Healthy	
		5 Apr 2022			−	NA	Healthy	
BKK-K1	Dog	4 Apr 2020	F	Indoor/outdoor	+, 31	NA	Pneumonia	No

a These animals and humans lived within the same colony or had contact with other SARS-CoV-2-positive animals.

b These animals lived within the same colony or had contact with other SARS-CoV-2-positive animals.

c These animals lived within the same colony or had contact with other SARS-CoV-2-positive animals.

d These animals lived within the same colony or had contact with other SARS-CoV-2-positive animals.

e This animal and human had contact with other SARS-CoV-2-positive animals.

f Respiratory symptoms present at the time of sample collection.

g History of contact with SARS-CoV-2-positive humans within 14 days prior to sample collection.

h *C_T_* values for the RdRp gene of SARS-CoV-2 determined by qRT-PCR are indicated.

i NA, no information available; M, male; F, female; +, positive; −, negative.

**TABLE 2:** Amino acid mutations of the SARS-CoV-2 spike protein obtained from cats and dogs in this study

SARS-CoV-2 strain	GenBank accession no.	Location	Species	PANGO lineage	Characteristic mutation												
					69–70 deletion	Amino acid substitution in spike protein											
						R346K	W353C	K417N	L452R	T478K	N501Y	A570D	H655Y	P681H	T716I	S982A	D1118H
Wuhan-Hu-1	NC_045512	China	Human	B	No	R	W	K	L	T	N	A	H	P	T	S	D
AFRIMS-COV1392-2021	MZ888536	Thailand	Human	B.1.1.7	Yes	R	W	K	L	T	Y	D	H	H	I	A	H
AFRIMS-COV2483-2021	MZ888555	Thailand	Human	B.1.1.7	Yes	R	W	K	L	T	Y	D	H	H	I	A	H
CU27042N	MZ396818	Thailand	Dog	B.1.1.7	Yes	R	W	K	L	T	Y	D	H	H	I	A	H
CU27081N	MZ401455	Thailand	Cat	B.1.1.7	Yes	R	W	K	L	T	Y	D	H	H	I	A	H
BKK-K1/2020	ON966106	Thailand	Dog	B.1	Yes	R	W	K	L	T	N	A	H	P	T	S	D
PK055/2021	ON966107	Thailand	Cat	B.1.617.2	No	R	W	K	R	K	N	A	H	R	T	S	D
BKK092/2021	ON966108	Thailand	Cat	B.1.1.7	Yes	K	W	N	R	K	N	D	H	H	I	A	H
BKK042/2021	ON966109	Thailand	Cat	B.1.1.7	Yes	R	W	K	L	K	Y	D	H	H	I	A	H
BKK100-08-21/2021	ON966110	Thailand	Cat	B.1.1	Yes	R	C	K	L	T	Y	D	Y	H	I	A	H
BKK108/2021	ON966111	Thailand	Cat	B.1.1.7	Yes	R	C	K	L	T	Y	D	Y	H	I	A	H
BKK102/2021	ON966112	Thailand	Cat	B.1.1	Yes	K	C	N	L	T	Y	D	Y	H	I	A	H
BKK100-08-11/2021	ON966113	Thailand	Cat	B.1.1.7	Yes	K	W	N	R	K	Y	D	Y	H	I	A	H
BKK-P1/2021	ON965806	Thailand	Human	B.1.1.7	Yes	R	C	K	L	T	Y	D	Y	H	I	A	H
BKK-P2/2021	ON965808	Thailand	Human	B.1.1.7	Yes	R	C	K	R	T	Y	D	Y	H	I	A	H
BKK-P3/2021	ON965807	Thailand	Human	B.1.1.7	Yes	R	C	K	L	T	Y	D	Y	H	I	A	H
BKK-ST10/2021	ON965809	Thailand	Cat	B.1.1.7	Yes	R	W	K	L	T	Y	D	H	H	I	A	H
BKK-ST023.1/2022	ON966114	Thailand	Dog	BA.2	Yes	K	W	N	L	K	Y	A	Y	H	T	S	D
BKK-ST023.8/2022	ON966115	Thailand	Human	BA.2	Yes	K	W	N	L	K	Y	A	Y	H	T	S	D

### Human-to-pet and reversed pet-to-human transmission of SARS-CoV-2.

Apart from SARS-CoV-2 surveillance in cats and dogs, we investigated two independent SARS-CoV-2 scenarios, so-called scenarios A and B, which presented zoonotic potential during the fourth and fifth waves of COVID-19 in Thailand. A thorough investigation with in-depth interviews of owners, veterinarians, and veterinary staff, together with sampling of neighboring animals and contacted caretakers, was initiated to identify potential transmission for environmental and occupational risk assessment.

**FIG 3:**
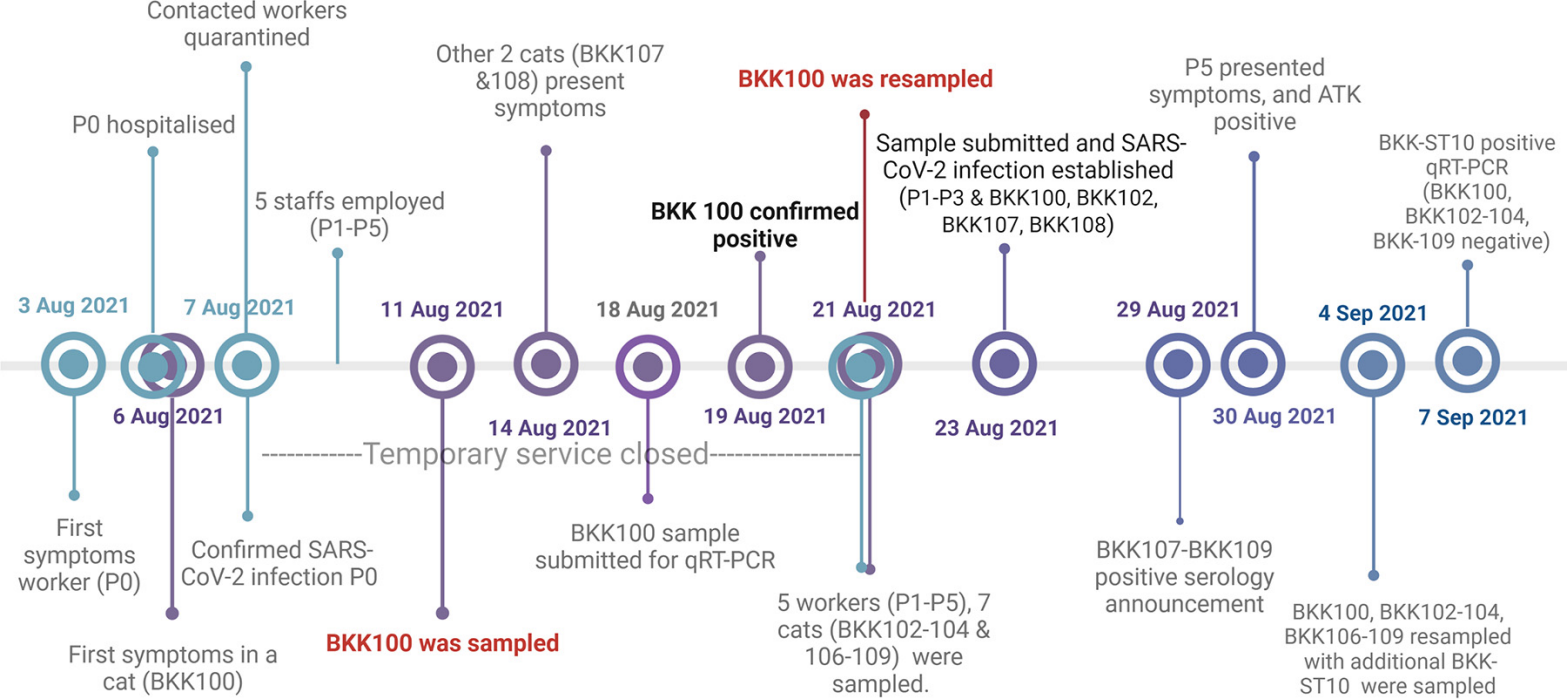
Schematic timeline of events of SARS-CoV-2 infection in scenario A. Dates are presented for the corresponding events. Symbols in turquoise, purple, and indigo indicate events affecting humans, cats, and both cats and humans, respectively.

For scenario A, we found a SARS-CoV-2-positive sample derived from a cat (BKK100) sheltered in a veterinary hospital in Bangkok, Thailand, at the beginning of August 2021 (Fig. 3). This cat had a history of contact with a veterinary nurse (P0) with SARS-CoV-2 infection. Cat BKK100 was a 9-year-old, indoor, domestic shorthair, feline leukemia virus (FeLV)-positive, castrated male, which had undergone treatment for chronic kidney disease. Cat BKK100 was sheltered separately in a unit with seven other healthy cats ranging in age from 4 to 12 years. This scenario began with a veterinary nurse (P0) who took care of this unit who presented with a fever and a runny nose on 3 August 2021. Subsequently, cat BKK100 showed respiratory symptoms (including sneezing, coughing, and ocular discharge) on 6 August 2021, the date on which caretaker P0 was hospitalized. After the announcement of COVID-19 in caretaker P0, other contacted caretakers were quarantined at home, and the veterinary hospital was cleaned, disinfected, and temporarily closed for 14 days. During the hospital’s service closure, five COVID-19-vaccinated staff members (P1 to P5) with evidence of SARS-CoV-2-negative tests and no contact history with caretaker P0 were separately rotationally employed to take care of BKK100’s unit from 8 to 21 August 2021. During this period, cat BKK100 still presented respiratory symptoms, and these respiratory signs were also detected in two other cats (BKK107 and BKK108). An oropharyngeal swab and blood of cat BKK100 cat were first sampled on 11 August 2021 and submitted for SARS-CoV-2 detection on 18 August 2021. The detection of SARS-CoV-2 in cat BKK100 with a *C_T_* value of 18 was confirmed on 19 August 2021, and the cat was resampled on 21 August 2021, together with the sampling of oropharyngeal swabs and blood collected from seven neighboring cats (BKK102 to -104 and BKK106 to -109). Based on the history, all caretakers wore only surgical masks and nitrite gloves for their protection when in contact with the cats. Nasal swabs were also obtained from caretakers P1 to P5 for SARS-CoV-2 detection, resulting in positive detection in cat BKK100, three neighboring cats (BKK102, BKK107, and BKK108), and three caretakers (P1 to P3), with varying viral copy numbers (Table 1). SARS-CoV-2-specific antibodies were detected in three cats (BKK107 to BKK109), and the results were announced on 29 August 2021. During quarantine, caretaker P5 presented symptoms and showed a positive COVID-19 antigen test kit (ATK) result on 30 August 2021; this event led to the resampling of cats BKK100 to -104 and BKK106 to -109, with the sampling of an additional cat (BKK-ST10) that lived nearby and had contact with caretaker P5 on 4 September 2021. Caretaker P5 refrained from providing a sample for SARS-CoV-2 tracing. Cat BKK-ST10 was positive for SARS-CoV-2 by qRT-PCR on 7 September 2021. The timeline regarding the investigation of scenario A is summarized in Fig. 3. Investigation of other caretakers and animals in this hospital revealed negative SARS-CoV-2 detection. After intensive management for COVID-19 according to CCSA guidelines for this hospital, there were no subsequent reports regarding the presence of SARS-CoV-2 infection in other animals and caretakers.

Scenario B presented during the fifth wave of COVID-19 in Thailand. We detected SARS-CoV-2 RNA in a 2-year-old, castrated male, indoor, singly raised, veterinarian-owned Yorkshire dog (BKK-ST023.1). The dog was healthy and accompanied the female veterinarian (BKK-ST023.8) to various clinics/hospitals located in Bangkok, where she worked. The dog was in contact with multiple clients and veterinary staff members at each place. Veterinarian BKK-ST023.8, despite having received triple vaccinations against COVID-19, presented a runny nose with fever on 24 March 2022. SARS-CoV-2 infection of human BKK-ST023.8 was confirmed by qRT-PCR. After positive testing, dog BKK-ST028.1 was tested by oropharyngeal swabbing daily from 25 March to 1 April 2022. SARS-CoV-2 RNA was detected from 25 March 2022 (*C_T_* = 29) to 29 March 2022 (*C_T_* = 38), with gradually increasing *C_T_* values, and veterinarian BKK-ST023.8 presented positive qRT-PCR results until 3 April 2022 (Table 1). Additionally, veterinarian BKK-ST023.8 had no history or risk of COVID-19 contact and no history of treatment of animals that presented respiratory disease within 7 days prior to the positive SARS-CoV-2 test results. Other humans with a history of contact with veterinarian BKK-ST023.8 reported the absence of SARS-CoV-2 infection.

### Phylogenetic relationship of SARS-CoV-2 from infected humans and related animals.

**FIG 4:**
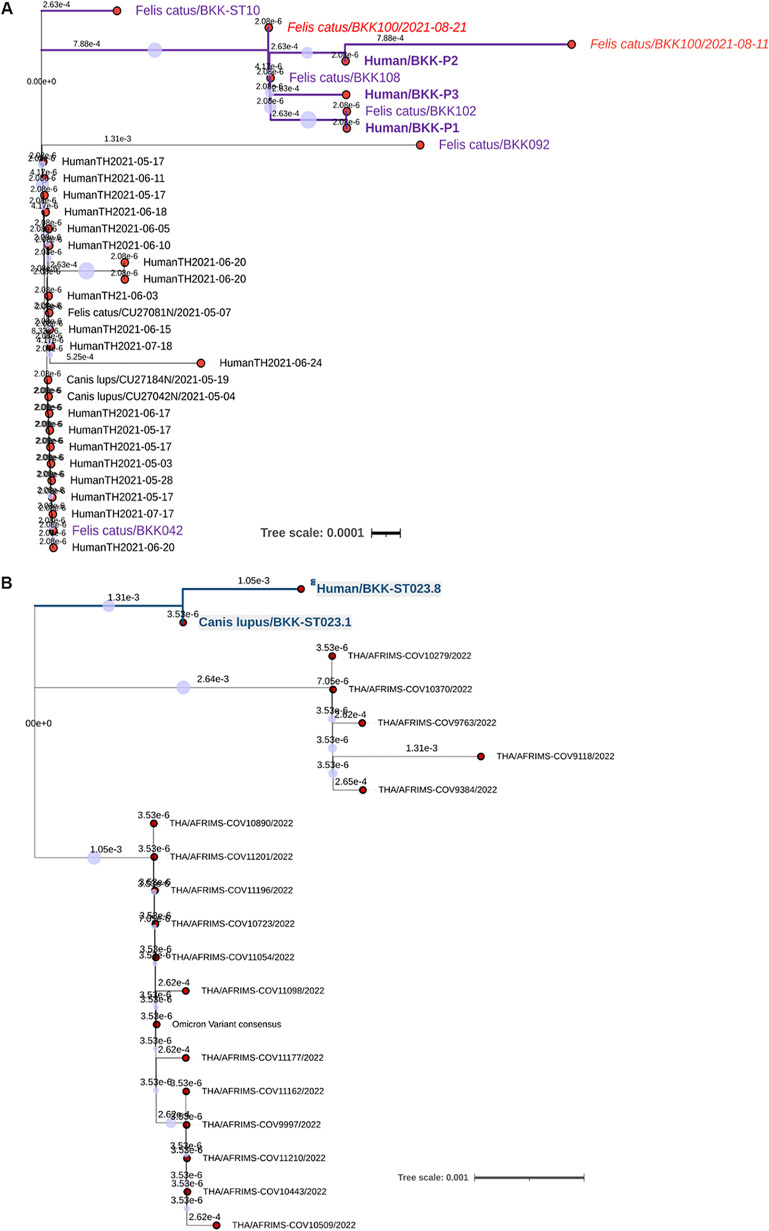
Phylogenetic analysis of animal-related human SARS-CoV-2 surface glycoprotein gene sequences detected in this investigation. Sequences derived from Alpha (B.1.1.7) (A) and Omicron BA.1 and 2 (B) VOCs indicated a sequence cluster between humans and animals. Sequences derived from cats and humans in scenario A are indicated in purple, and the red labels indicate sequences obtained from cat BKK100 at different sampling times. Sequences obtained from infected dog BKK-ST023.1 and veterinarian BKK-ST023.8 are indicated in light green. The scale bars indicate units of substitutions per site in each tree.

For scenario A, we characterized two complete SARS-CoV-2 genome sequences (BKK100/2021-08-11 and BKK100/2021-08-21) obtained from cat BKK100 at different sampling times using NGS. Genetic sequencing of the two collected samples revealed disparity mainly within the S gene. These results were similar to and in accordance with the results of conventional Sanger sequencing of the S gene using conventional RT-PCR amplification. Amino acid mutations between the two samples from cat BKK100 included K346R, W353C, N417K, R452L, and K478T. Whereas the sequence derived from the first sample collected from cat BKK100 (BKK100/2021-08-11) revealed more divergence, the second sample (BKK100/2021-08-21) showed genetic sequence similarity to the sequences derived from humans and other cats. All human sequences (P1 to P3) were nearly identical and clustered with the cat-derived sequences, presenting three specific nucleotide mutations (c.1050G>C, c.1233A>G, and c.1954C>T), and they were distinct from those of other Alpha VOC sequences obtained from humans and animals in Bangkok at the same time, presenting the amino acid changes W353C and H655Y (Fig. 4A). Synonymous mutations were also identified in SARS-CoV-2 obtained from cat sequences compared with the human sequences detected in Thailand. Among them, a diversifying mutation, L449R, was observed, which was found in sequences derived from the first samples collected from cat BKK100 (BKK100/2021-08-11) and human P2 but not in other derived sequences.

Regarding scenario B, whole-genome sequencing of SARS-CoV-2 indicated a close genetic relationship between the sequences from dog BKK-ST023.1 and veterinarian owner BKK-ST023.8, presenting a specific amino acid mutation, L941S, that resulted in genetic clade distinction from other Omicron variants found in humans living in Bangkok and other provinces in Thailand (Fig. 4B). Changes in amino acid mutations found in SARS-CoV-2 sequences detected in human BKK-ST023.8 and dog BKK-ST023.1 were compared with other Omicron VOC sequences found in humans in Thailand and dogs and cats from around the world. This study found specific amino acid signatures of animal SARS-CoV-2, which were not present in the sequences reported in humans in Thailand, in the sequence obtained from human BKK-ST023.8, including D339G, L371S, P373S, and F375S, similar to the sequence obtained from dog BKK-ST023.1.

## DISCUSSION

The present study involved a cross-sectional investigation of SARS-CoV-2 in cats and dogs presenting to animal hospitals/shelters from late March 2020 to June 2022 and traced back to the samples collected in the beginning of November 2019 to indicate the short survey prior to the announcement of emerging SARS-CoV-2. We identified the presence of SARS-CoV-2 in cats and dogs, even though there was a relatively low rate of detection of viral RNA due to possible reasons such as low viral titers in infected pets, a short duration of shedding (27), or local infection, as most positive cases were asymptomatic (28, 29). However, detection was prevalent in correspondence with SARS-CoV-2 pandemic waves in Thailand and was enriched in the fourth wave of the COVID-19 pandemic. Previous studies presented evidence of cats and dogs in Thailand being infected with the SARS-CoV-2 Alpha and Delta VOCs (30, 31); in the present study, we extended information regarding the detection of various SARS-CoV-2 VOCs in pets, demonstrating that various variants of SARS-CoV-2 had been circulating in these populations and indicating susceptibility to infection in these animals. The rate of susceptibility to each VOC in pets could not be determined from this study due to passive sampling; however, the Alpha VOC was relatively more prevalent in this investigation due to its endemicity in kenneled cats of scenario A, even though these infected cases were sampled during the fourth wave of the COVID-19 outbreak in Thailand when the Delta VOC (B.1.617.2) was predominant (32). Remarkably, the presence of SARS-CoV-2 could be related to the sampling time, the health condition of infected pets, and contact between pets and owners, as indicated by previous studies (10, 33). In accordance with the results of several studies (34, 35), we found that infected pets were cared for by SARS-CoV-2-infected owners residing in close contact with their pets, becoming prone to human-to-animal transmission. On the other hand, animal-to-animal infections have also been reported (9, 28, 36), indicating a potential source of SARS-CoV-2 transmission from infected animals to susceptible contacts, which we observed for such contagious infections in multiple cats in scenario A. SARS-CoV-2 spillover from infected animals back to humans has been evidenced in SARS-CoV-2 outbreaks on mink farms (11, 12).

Even though the rate of transmission of infection from animals back to humans is considered to be relatively low and often occurs in cases of close contact, we present evidence of spontaneous SARS-CoV-2 transmission in a cat colony and spillover back to humans. The nearly identical SARS-CoV-2 genome sequences obtained in duplicate from both NGS and Sanger sequencing with a specific amino acid signature in the S gene obtained from cats and contacted caretakers, together with the overlapping timeline of animal and human infections, suggested that the infections among them were epidemiologically related. Most substitutional variants present at the RBD positions were found in this study, and as interactions of the RBD and ACE2 receptors resulting in viral entry have been reported as key determinants of host susceptibility (37, 38), the RBD variants are thus of concern, as these mutations are associated with diverse susceptible hosts. After the initial detection of SARS-CoV-2 in cat BKK100, six other cats (three positive for SARS-CoV-2 RNA and another three positive for antibodies against SARS-CoV-2) and four out of five related caretakers, who initially tested negative for COVID-19 prior to contact with the infected cat BKK100 and did not have contact with the infected human P0 would later become infected with SARS-CoV-2, indicating possible contact with the SARS-CoV-2-infected cat and a possible risk for SARS-CoV-2 infection in this scenario. Although cat BKK107 was positive for SARS-CoV-2 by qRT-PCR, genetic sequencing could not be performed due to a very high *C_T_* value. A case of cat-to-human SARS-CoV-2 infection was recently reported, assuming transmission from a cat to a veterinarian due to sneezing during physical examination (39), suggesting the possible spread of infection from the infected cat to the susceptible human via close contact. Once infection in scenario A occurred, subsequent spread to other kenneled cats and caretakers as they were living in a room under the same environmental conditions was highly possible. According to the CCSA regimen for the prevention of SARS-CoV-2 spread, human P0 was isolated, and the fomites were immediately disinfected after confirmed infection, inhibiting the collection of samples from the household equipment, which was a limitation of this investigation.

Further evidence that suggested that cats in scenario A served as a potential source of SARS-CoV-2 infection in such scenarios was supported by the distinct phylogenic topologies of SARS-CoV-2 sequences obtained from the studied animals, related humans, and other humans in the same geographic area. Phylogenetic analysis showed that SARS-CoV-2 sequences obtained from cats and humans were deeply clustered and were distinct from sequences obtained from other infected humans in the same province at the same time, indicating a lower possibility of external transmission from other infected cases. Regarding scenario B, although we could not determine the potential source of primary infection, specific amino acid signatures previously described for the SARS-CoV-2 Omicron VOC (40) isolated from infected dogs were present in the SARS-CoV-2 sequence obtained from owner BKK-ST023.8. Together with the overlapping timeline and in-depth interviews, this finding indicates an epidemiological interaction between dog BKK-ST023.1 and owner BKK-ST023.8. Tracking people or animals who came into contact with this dog prior to SARS-CoV-2 detection in this owner was not possible, so the question of whether the dog or the owner was the primary source of infection remains unanswered. Because SARS-CoV-2 RNA was detected in such a relationship, the timeline of detection was longer for owner BKK-ST023.8 than for her dog; the potential source in this scenario that arose from dog BKK-ST023.1 also warranted further supporting evidence.

Interestingly, we found genetic disparity between two samples collected at different time points from cat BKK100 that had experienced chronic FeLV infection and suffered from kidney disease, suggesting the within-host evolution of SARS-CoV-2, which has been frequently observed in SARS-CoV-2 infection of immunocompromised patients (41–43). Thus, intrahost mutation of SARS-CoV-2 in immunocompromised cats is possible, as indicated by a previously reported observation of a SARS-CoV-2-infected immunocompromised cat (44). Furthermore, the rapid adaptation of SARS-CoV-2 variants to nonhuman mammalian hosts after initial infection with the original virus was reported (45), highlighting the potential of reinfection of humans with new variants arising in such animals that have close contact with humans.

The primary source of infection by SARS-CoV-2 in these two scenarios could not be determined in this study; however, SARS-CoV-2 transmission from humans to animals and from animals to animals and reversed transmission from animals to humans, especially in pet animals that have shared a habitat and have had close contact with humans, are indicated, which should not be underestimated and needs further observation.
